# Influence of Antipsychotic and Anticholinergic Loads on Cognitive Functions in Patients with Schizophrenia

**DOI:** 10.1155/2016/8213165

**Published:** 2016-04-10

**Authors:** Michael Rehse, Marina Bartolovic, Katlehn Baum, Dagmar Richter, Matthias Weisbrod, Daniela Roesch-Ely

**Affiliations:** ^1^Department of Psychiatry, University of Heidelberg, 69115 Heidelberg, Germany; ^2^Psychiatrisches Zentrum Nordbaden, 69168 Wiesloch, Germany; ^3^Department of Psychiatry and Psychotherapy, SRH Hospital Karlsbad-Langensteinbach, 76307 Karlsbad, Germany

## Abstract

Many patients with schizophrenia show cognitive impairment. There is evidence that, beyond a certain dose of antipsychotic medication, the antipsychotic daily dose (ADD) may impair cognitive performance. Parallel to their D2 receptor antagonism, many antipsychotics show a significant binding affinity to cholinergic muscarinic receptors. Pharmacological treatment with a high anticholinergic daily dose (CDD) significantly impairs attention and memory performance. To examine the relationships between individual cognitive performance and ADD and/or CDD, we conducted a retrospective record-based analysis of a sample of *n* = 104 in patients with a diagnosis of schizophrenia, all of whom had completed a comprehensive neuropsychological test battery. To calculate the individual ADD and CDD, the medication at the time of testing was converted according to equivalence models. After extracting five principal cognitive components, we examined the impact of ADD and CDD on cognitive performance in the medicated sample and subgroups using multiple regression analysis. Finally, locally weighted scatterplot smoothing (Loess) was applied to further explore the course of cognitive performance under increasing dosage. Results showed significant negative effects of ADD on performance in tests of information processing speed and verbal memory. No effects were found for CDD. The potential neuropsychopharmacological and clinical implications are discussed.

## 1. Introduction

Besides the familiar positive and negative symptoms, cognitive symptoms constitute an important symptom dimension of schizophrenia. Many patients present cognitive deficits in domains such as attention, memory, and various subtypes of executive functions [1]. Cognitive symptoms appear at the time of or even before the appearance of positive symptoms [2] and remain relatively stable during the course of the disease [3]. One of the most important findings is that, in contrast to positive symptoms, cognitive symptoms are associated with functional outcome, that is, how well patients integrate socially and at work. Executive function, verbal memory, and vigilance, in particular, appear to be the best predictor variables for functional outcome [4]. The rate of employment among patients in Europe with schizophrenia is estimated to range within 8–35% [5], which demonstrates the high economic and social burden of the disease. Patients with better cognition are more likely to be in full- or part-time employment within two years of diagnosis [6]. These findings indicate the importance of cognition for the integration of patients into the community.

Due to their negative impact on functional outcome, the treatment of cognitive deficits has become a focus for research. There is evidence that antipsychotic treatment may have a small positive effect on cognition [7, 8]. The postulated advantage of second-generation over first-generation antipsychotics has not been confirmed, however, for either chronic patients in the CATIE Study [9] or first episode patients in the EUFEST Study [10]. There is also evidence that much of the performance improvement in cognitive assessments found in longitudinal studies may result from practice effects [11].

Rather than positive effects, antipsychotics may have adverse effects on cognition. First-generation antipsychotics have been shown to impair procedural learning and memory, especially at high doses [7, 8]. There is also evidence for a strong association between high doses of mono- or polypharmacy and significant impairment in cognitive performance [12], although some research has failed to replicate these findings [13]. Furthermore, antipsychotics may contribute to brain tissue loss when prescribed for a long time and at high dosages [14].

Two mechanisms may explain cognitive dysfunction under antipsychotic medication. One is the dopamine receptor blockade. In addition to its beneficial effects on the positive symptoms of schizophrenia (treatment of abnormal salience in delusions, focusing of thoughts, etc.), numerous studies have shown a correlation with impaired cognition in some circumstances [15, 16]. The cortical-striatal-thalamo-cortical loop model suggested by Alexander et al. [17] postulates direct and indirect pathways that in healthy subjects modulate cognitive processing originating from dopaminergic receptor transmission. Unbalanced dopamine receptor blockade leads to significantly less striatal and telencephalic activity when cognitive tasks are performed as an indicator of impaired cognitive functioning, the most significant effects being on motor speed and attention [18]. Individual variations in vulnerability to these mechanisms might be indicated by different endogenous baseline dopamine levels and varying turnover rates, resulting in impairment in some subjects while improving cognitive performance in others [19]. Another study showed in a single blind design that in healthy subjects a subchronic dosage (7 days) of antipsychotic medication had a negative impact on speed of information processing, attention, and learning compared with a placebo [20].

A second mechanism to explain cognitive dysfunction under psychotropic medication used in the treatment of schizophrenia is the effect of cholinergic blockade. This explanation is supported on a biological level by the observation of a drug-specific binding profile to the cerebral muscarinic receptors found in nearly all known cholinergic systems of the brain. In particular, parietal and frontal cortical projections of the nucleus basalis are affected, with declarative memory and complex attention being mainly impaired by high anticholinergic daily doses (CDD) [21]. The aforementioned circuits lose their ability to normatively modulate signal influx and ultimately cognitive functioning when under excessive load [22]. Moreover, one study showed that patients with schizophrenia profited less from cognitive training when the serum anticholinergic load before training was high [23].

To summarize, antipsychotics have only a small clinically relevant effect on cognition and there is some evidence that exceeding a certain level of antipsychotic daily dose (ADD) may impair cognitive function and facilitate brain tissue loss in some patients. Similarly, most studies have found that a high CDD is associated with impaired cognitive functioning. Additionally, substances that stimulate dopamine and acetylcholine can improve cognition, so an optimum balance of these neuromodulators is necessary for normal cognitive functioning.

In the light of these findings, our study aimed to evaluate, using a retrospective and record-based design, the influence of ADD and CDD loads on specific cognitive performances of patients with a diagnosis of schizophrenia who had undergone a comprehensive neuropsychological routine. Furthermore, the specific equivalent doses at which cognitive performance started to fall beneath the patients' baseline levels were assessed by applying Loess analyses.

Unlike the studies cited above, which in the main assessed the influence of ADD and CDD separately, our study examined the influence of both ADD and CDD on patients' performance on a well-evaluated cognitive test battery. Differences in and interactions between ADD and CDD effects on cognition could therefore be evaluated simultaneously. Our study also included a relatively large sample of patients with a heterogeneous pharmacological regime, thereby mirroring typical day-to-day prescription practice.

## 2. Materials and Methods

### 2.1. Experimental Procedures

Data were collected from a pool of 458 psychiatric patients who between the years 2004 and 2010 had undergone comprehensive neuropsychological testing in two-hour morning sessions as part of clinical routine at the Psychiatric Outpatient Unit for Cognitive Training of the Psychiatric Department at the Heidelberg University Hospital, Germany. The patients had taken a battery of neuropsychological tests based on the recommendations of MATRICS for measuring the cognitive domains deficient in schizophrenia [24]. Patients had given written informed consent for the evaluation of the data for scientific purposes. The use of the data was approved by the Ethics Commission of the Faculty of Medicine of the University of Heidelberg.

Out of the pool of 458 patients, *n* = 126 fulfilled the criteria for an ICD 10 diagnosis of schizophrenia (F20.0) confirmed by records at discharge. Patients with clinically diagnosed schizoaffective disorder (F25.X) were excluded from the trial. For the demographic and clinical characteristics of the sample, see Table 1. As standardized clinical tests such as the Positive and Negative Symptoms Scale (PANSS) were not available for our sample, to control for illness severity we included time since onset of illness. This parameter is known to predict the outcome of individual functioning for chronic schizophrenia patients [25]. A prerequisite for being eligible for testing was that patients were in nonacute stages of the disease, so the confounding effects of acute symptoms on cognition were minimized.

The neuropsychological constructs were divided into declarative verbal memory (Verbal Learning and Memory Test/*Verbaler Lern- und Merkfähigkeitstest*), complex verbal tasks (Wechsler Memory Scale Text Reproduction/*Logisches Gedächtnis* and Regensburg Word Fluency Test/*Regensburger Wortflüssigkeits-Test*), attention (the d2 Test of Attention/*Test d2 Aufmerksamkeits-Belastungs-Test *and Test of Attentional Performance (TAP)/*Die Testbatterie zur Aufmerksamkeitsprüfung* Vigilance subtest), information processing speed (TAP and the Trail Making Test (TMT-A)), and executive functioning (TMT-B, TAP Flexibility subtest) [26–30].

At the time of testing, 104 out of 126 patients were receiving psychopharmacological treatment with either mono- or polypharmacy with known antipsychotic and/or anticholinergic effects. For inpatients only, compliance wa**s** monitored by serum levels during the hospital stay. The ADD was transposed into risperidone equivalents based on “Model 2” [12] for transposing dose equivalents of first-generation antipsychotics to second-generation antipsychotics (50 mg chlorpromazine eq = 1 mg Haloperidol eq = 1 mg Risperidone eq), based on the mean modal doses of the CATIE trials and the chlorpromazine equivalent recommendations of the Patient Outcomes Research Team (PORT) [31]. In the latter study, the individual daily doses correlated closely with the individual risk of developing extrapyramidal side effects measured by the Simpson Angus Scale.

The CDD, on the other hand, was expressed through benztropine-mesylate equivalents (BZT-Eq), as the benztropine mesylate equivalent dose correlates highly with the risk of developing anticholinergic side effects [32]. The derived anticholinergic potency predicted poorer cognitive performances in complex attention tasks and memory [21]. Benztropine mesylate as an equivalent dose unit has the inherent advantage that its receptor binding and pharmacodynamic characteristics are well known. Additionally, its cholinergic binding potential is within the same range as other anticholinergics such as atropine. Based on these considerations, we calculated the individual CDD of every case using the daily BZT-Eq (see Table 2).

### 2.2. Statistical Analyses

Firstly, we conducted a principal component analysis (PCA) to reduce the number of variables by combining our indicators with a few representative factors. Since a complete dataset is necessary for executing a PCA, we first considered options for handling the missing data. Listwise deletion would have reduced the number of observations to an unacceptable level. Mean imputation methods, although widely used, are not recommended from a statistical perspective [33]. After reviewing relevant literature [34, 35] and verifying the randomness of the missing data using Little's MCAR test, we found the Maximum Likelihood (ML) procedure using Expectation Maximization (EM) to fit best the analysis requirements for our relatively large sample. After eliminating extreme outliers, testing the dataset for normal distribution, and applying ML, sample size could be preserved and principal component analyses were computed until viable factor solutions were obtained.

The main analysis focused on several multiple regression models with the factor values serving as criteria to examine the effects of increasing pharmacological load on cognitive performance. Initially, we applied both quadratic and linear models. The linear approach appeared viable, whereas the quadratic function showed no relevant effects. We discarded quadratic approaches and conducted further analysis using linear regression. As 50% of patients did not receive additional anticholinergic medication and approximately 17% received no medication, in addition to analyzing the sample as a whole we also analyzed subgroups of patients (Group A, ADD receivers, and Group B, ADD + CDD receivers) in order to better differentiate the medication effects. Out of the original 126 patients, 22 subjects received no medication, while for the remaining medicated sample (*N* = 104) 50% received monotherapy (one antipsychotic medication only, *N* = 52) while the other half received polypharmacy (defined as more than one antipsychotic and/or other psychopharmacological classes). Distribution in the subgroups (see below) was also fairly even: in Group A (*n* = 41) 24 patients (58,5%) were on monotherapy versus 17 patients (41,5%) on polypharmacy. In Group B (*n* = 63), 28 patients (44,4%) were on monotherapy versus 35 patients (55,5%) on polypharmacy. Furthermore, mean ADD for the whole sample was 5,35 mg risperidone equivalents (with 6,84 mg for Group A and 6,25 mg for Group B), while mean CDD was 5,17 mg benztropine equivalents (with per definition 0 mg equivalents for Group A and 10,25 mg for Group B).

To explore further the findings from multiple regression analyses, the last step consisted of applying Loess [36, 37]. (It is important to note that Loess is a descriptive method and does not imply causality or allow deduction.) This was applied firstly to the whole sample (ADD and CDD receivers) and secondly to the subgroups, while interpreting the course of cognitive performance in different principal components under increasing loads. Raw values of performance in cognitive domains were first transformed to their corresponding *z*-values and converted to their natural logarithm value for more homogenous scatterplot distribution. Point recognition for curve smoothing was set to 65%. All statistical analyses were computed using SPSS 20.

## 3. Results

### 3.1. Principal Component Analysis

We conducted several principal component analyses to reduce the variable set into a few broader meaningful cognitive factors. We selected oblimin rotation and thus allowed dependence between factors, since an assumption of independent cognitive domains does not accord with current cognition theory. Within the framework of the initial PCA, consisting of the complete variable set, the Verbal Learning and Memory Test results showed high loadings on several factors, complicating their interpretation. Since verbal memory is an important domain of cognitive deficits in schizophrenia, we excluded the variables from the overall PCA because of the difficult loading pattern. However, we conducted a second PCA consisting solely of all verbal memory indicators in order to reduce the number of test parameters and to obtain fewer and more reliable factor scores. Indeed, the verbal memory test parameters appeared to converge tightly into one broad factor explaining approximately 75% of the total variance (see Table 3(a)). This composite verbal memory component was then treated as* declarative verbal memory *(VM). In the main PCA on the other hand, we were able to extract four factors representing different cognitive domains, explaining approximately 65% of the total variance. The factor loadings are displayed in Table 3(b) labelled* complex verbal tasks *(CVT),* information processing speed *(IPS)*, executive functioning *(EF), and* attention *(ATT), but some ambiguity remains in their interpretation. The intercorrelation of the four factors is displayed in Table 3(c).

### 3.2. Baseline Cognitive Performance

Mean percentile ranks (PR) of control population (from available test norms, see test references) were used to compare the performances within our sample. At a descriptive level overall baseline performance of our sample showed that for all parameters measured patients performed worse than expected for a control population (PR < 50). Performances of patients in subgroups A and B were similar; unmedicated patients tended to perform better (values for the subsamples can be required from corresponding author). For some cognitive variables no percentile ranks were available or the missing data was high, so these were not presented; see Table 3(d) for the mean percentile ranks.

### 3.3. Effects of ADD and CDD

Finally, the effects of ADD and CDD loads on the different cognitive domains were analyzed step by step using linear multiple regression models in different subsets of the initial sample, all controlled for age, gender, education, and duration of illness. The analytical process, including the results, is described in detail in the following paragraphs. For this step of the analyses, only medicated patients (*n* = 104) were included.

*Multiple Regression Analyses in the Medicated Sample (n = 104).* A significant effect of ADD on IPS (*B* = .242, *p* < .05) was found. No significant effects of CDD on any cognitive domain were found.

*Multiple Regression Analyses in the Subgroups of ADD Only versus ADD + CDD Receivers. *We then split the patients into subgroups: Group A, those who had received antipsychotics without any anticholinergic effects (ADD only; *n* = 41), and Group B, all those who had received antipsychotic medication with anticholinergic properties and/or additional medication with anticholinergic properties, except for two patients who received CDD without ADD (ADD + CDD; *n* = 63). We first compared these two groups using ANOVAs and contingency table analyses. No significant group differences could be observed in demographic, clinical, or cognitive variables. Tendency effects could be observed for age (*p* < .10) and time since onset of illness (*p* < .10). For a summary of demographic and clinical characteristics of the subgroups, see Table 4. Finally, we analyzed possible medication effects for Group A (ADD only receivers) and Group B (ADD + CDD receivers) separately using multiple regression analyses, including (in both groups) ADD, CDD, and the demographic/clinical control factors.

In Group A (ADD only, *n* = 41), no significant results were found for any of the cognitive factors if all control variables were included. However, in the preliminary correlational analysis for Group A, VM correlated significantly with ADD (*r* = −.344, *p* < .05), so the missing effect in the full regression model could be due to poor statistical power. In more parsimonious regression models, the significance of the ADD effect on VM in Group A ranges between *p* values of .10 and .01, depending on which control variables are included. The best parsimonious regression model, explaining approximately 19% of variance and including only gender as a control variable, showed a significant ADD effect on VM (*B* = −.325, *p* < .05). No effect was found for CDD on any cognitive domain.

In Group B (ADD + CDD, *n* = 63), a significant effect of ADD on IPS could be observed (*B* = .292, *p* < .05). Additionally, we found a tendency effect of CDD on EF (*B* = .280, *p* < .10).

**Figure 1:**
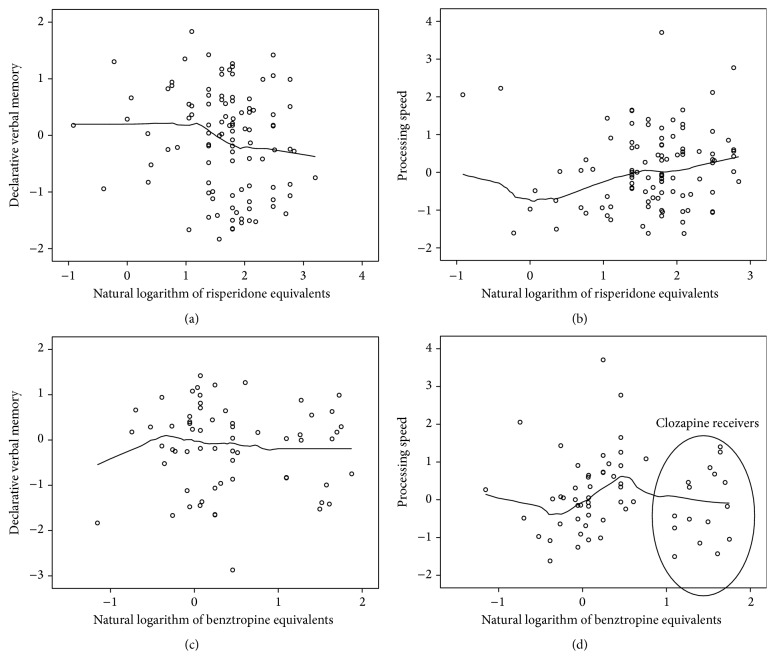
Locally weighted scatterplot smoothing (Loess) plot of cognitive performance in declarative memory and processing speed under escalating antipsychotic and anticholinergic daily dose.

*Locally Weighted Scatterplot Smoothing (Loess). *To explore further our findings from the multiple regression analyses, we applied Loess to examine the courses of cognitive performance under increasing equivalent doses within the whole sample and the subgroups. In most cases (ATT, CVT, VM, and EF), the courses of cognitive performance did not yield particularly revealing results. Nevertheless, looking at the whole medicated sample (*n* = 104), verbal memory performance initially increased slightly, peaked, and then decreased and dropped below its sample mean when dosage exceeded 4.53 mg/d RIS-Eq. Additionally, IPS performance in Group B (ADD + CDD receivers) showed that while increasing anticholinergic doses initially worsened results, a group of patients receiving very high anticholinergic doses (BZT-Eq > 20 mg/d) achieved much better results. Interestingly, all those patients received clozapine as their main antipsychotic medication (see Figure 1(d)).

## 4. Discussion

The aim of our study was to examine cognitive performance in relation to pharmacological daily doses of antipsychotic and anticholinergic medications and their combination within specific cognitive domains, namely, declarative memory, information processing, executive function, and attention. As expected for patients suffering from schizophrenia the baseline cognitive performance for all variables was below the one expected for a control population. At a descriptive level patients without medication tended to perform better than those with medication, although still impaired in many different tasks.

We found that higher ADD was significantly associated with slower information processing. When examining subgroups (antipsychotic with and without anticholinergic properties), we found that patients receiving antipsychotic medication with anticholinergic effects (such as olanzapine, Group B) had significantly slower information processing speed under increasing load. This result was not present in patients receiving antipsychotics without an anticholinergic binding profile (such as aripiprazole, Group A). These results imply that the additional anticholinergic load to antipsychotics contributes to slower information processing in schizophrenia.

Another significant negative effect of ADD on declarative memory was found in Group A. Nevertheless, the interpretation of this effect, which is oscillating in strength and significance level depending on the number and type of predictors included, is not completely clear, in contrast to the robust significant effect of ADD on information processing speed. The effects of medication on attention, complex verbal tasks, and executive functioning found in other studies could not be reproduced.

These results are partially in line with Élie et al. 2010 [12], who also found declarative memory to be impaired by increasing ADD. Similarly another study [38] showed for polypharmacy a negative correlation of cognitive scores with the use of antipsychotic medication. Along similar lines, a study [39] found polypharmacy and/or excessive dosage (chlorpromazine equivalents of 1000 mg/d) to be associated with poorer performance on visual memory, delayed recall, Intelligence Quotient (IQ), and executive function. Dose reduction was associated with improvements in cognitive functioning [40]. One study [13] found no differences in cognitive scores between patients with schizophrenia taking excessive daily doses of chlorpromazine equivalents (>1000 mg/d) and patients taking “normal doses” (<1000 mg/d CPZ-E), but their results may be confounded by their definition of “normal dose,” which was approximately 500 mg/d CPZ-E. According to Model 2 of Élie et al. [12], this equates to 10 mg Haldol or 5 mg risperidone equivalent, a dosage that they and we (see below) found to begin to be cognitively disadvantageous.

The deleterious effects of anticholinergic properties on cognition in patients with schizophrenia are well known. There is strong evidence that a higher anticholinergic load impairs cognition, especially verbal memory and attention, and there is improvement in memory tasks under decreasing anticholinergic medication doses [41, 42]. The adverse effects of anticholinergic load are more prominent in patients with a higher risk of cognitive impairment (e.g., patients with neurological diseases or psychiatric disorders or the elderly). The deleterious cognitive effect of long-term use has been recently reviewed [43]. We also expected to find higher levels of cognitive impairment under increasing CDD load. No significant effects were found under increasing CDD load in the whole sample, or in the subgroups. This may be because equivalent dose calculations based on Minzenberg et al. were insufficiently precise due to the more diverse pharmacological regimes in our sample and/or insufficient data for anticholinergic equivalent doses for some drugs. Given these considerations, our finding of a trend effect on EF under increasing CDD is likely to be coincidental, especially as it could not be reproduced for CDD in the whole sample or in Group A.

Further, in line with Élie et al., we analyzed cognitive performance using Loess and found the same negative trend in cognition after splitting the sample into the subgroups and calculating performance in different domains. In Group B (ADD + CDD receivers) information processing speed was impaired by increasing CDD. Additionally, in the whole sample, verbal memory performance was impaired by increasing ADD when dosage exceeded 4.26 mg Ris-Eq.

Surprisingly, the “highest-dose” CDD group (BZT-Eq > 20) achieved relatively better information processing results than the “moderate-dose” group (0 < BZT-Eq < 15). The highest anticholinergic doses were exclusively from the administration of clozapine, which contains a high affinity to 5-HT and dopamine receptors of the D4 type and NMDA agonism, as well as extensive anticholinergic potential (M1, M2, and M3) and agonistic M4 properties. While clozapine has been repeatedly reported to produce mild improvements in accuracy and executive function at therapeutic doses [44, 45], at high equivalent doses we expected the anticholinergic load to impair information processing as much as any other cognitive domain. Serum anticholinergic activity is proved to be higher for clozapine than risperidone [46] or olanzapine [47]; but there are no differences in the detrimental effect on global cognition, as measured by the MMSE Scale. Our findings of a lack of detrimental cognitive effects irrespective of the higher anticholinergic load of clozapine are therefore in accordance with those studies, although the tests used to assess cognitive function (MMSE versus a broad neuropsychological battery) were not equivalent. It has yet to be determined if the very broad receptor binding profile of clozapine (a “dirty drug”) without strong D2 receptor affinity compensates for the detrimental impact of anticholinergic effects on cognition. The cortical-striatal-thalamo-cortical loop model of dopamine-dependent cognitive modulation [17] could indicate that less dopaminergic imbalance would lead to comparatively better cognitive performance. Moreover, it can be further speculated whether the potential cognitive enhancing properties of clozapine's NMDA agonism [48] and M4 agonism [49] may compensate for the detrimental effects of anticholinergic activity.

To summarize, our results reinforce the clinical importance of an appropriately set psychopharmacological medication plan, as even moderate doses of antipsychotic medication may impair cognitive functioning. Although these effects may vary between patients, the rationale should be borne in mind when prescribing medication. While a higher initial dose of antipsychotic medication is a valuable tool for attenuating acute symptoms such as delusions and hallucinations, relapse-preventing medication should be chosen taking into account its potential impact on cognition and on functional outcome. As shown in the EUFEST Study [10], when dosage of antipsychotics is kept at the normal-low range and polypharmacy avoided, a small positive effect on cognitive function can be detected. Furthermore, the results of our study strengthen the evidence for the deleterious effect of medication with an additional anticholinergic binding profile. We argue therefore that in nonacute phases of the disease the pharmacological treatment plan should include the minimum dosage of antipsychotic medication necessary and should avoid add-on use of anticholinergics.

The limited evidence for clinically relevant effects of antipsychotics on cognition and functional outcome has led to increasing interest in other treatments for cognitive deficits. Cognitive training/remediation has been shown to be effective in improving cognition and functional outcome, especially as part of multimodal rehabilitation programs [50, 51]. Cognitive enhancers, many of which stimulate acetylcholine, and catecholamines, such as dopamine, are promising but still controversial [52]. The combined use of cognitive remediation and pharmacological cognitive enhancement has been recommended [53] but has not been systematically studied as yet and would merit further investigation.

The fact that we found adverse effects only for information processing speed and verbal memory, but not for attention and executive functions, could indicate that rather specific cognitive functions are being impaired, rather than cognitive performance in general, and that the effects may differ for ADD and CDD. Nevertheless, both information processing speed and verbal memory are highly related to functional outcome [54]. These functions are* per se* disturbed in schizophrenia. If they become more impaired through excessive medication dosage and additional anticholinergic use, this constitutes an even greater barrier to treatment of patients for such deficits, for example, through cognitive remediation [23], that would facilitate their subsequent reintegration into the community.

There are limitations to our study. Firstly, the results must be interpreted carefully because the analysis is based exclusively on retrospective correlative data, which means that the causality of relationships cannot be determined. Furthermore, our sample consisted of patients at a younger age and short illness duration (21.2 months). Both factors contribute to a tendency of better cognitive performance at MATRICs related tasks [55]. Also, we excluded patients with clinically diagnosed schizoaffective disorder, since neurocognitive functioning in schizoaffective disorder differs from that in schizophrenia [56]. Also the rate of polypharmacy versus monotherapy was balanced and the relatively low number of clozapine receivers (*N* = 13) is conformed to the young age of the sample. The gender ratio with male predominance is similar to other schizophrenia studies; additionally, gender was controlled for in the analyses. Subgroup B has been ill for a longer time but our data does not confirm that they were obviously on more polypharmacy (55,5% versus 44,4% on monotherapy) and did not have higher risperidone-equivalent dosages but had per definition more benztropine equivalents. These characteristics have to be taken into account when comparing the results with other studies. Nevertheless the present results offer a foundation for future prospective and experimental designs which could throw further light on the possible causal effects of antipsychotic medication on cognitive functioning and its impact on functional outcome. Secondly, the large number of missing data had to be handled by applying a Maximum Likelihood (ML) method. Although this is a more adequate method than traditional techniques, it is based on a number of assumptions, two major ones being multivariate normality (as in the case of multiple regression analysis) and MAR (missing at random) data. We tested our data for missing completely at random (MCAR) and examined variable distributions. Whereas our data fulfilled the MAR assumption (by fulfilling MCAR), this was not the case for multivariate normality. We did not need to perform a specific multivariate normality test as several of the single variable distributions already showed significant deviation from normality. The lack of multivariate normality, coupled with a high amount of missing data, means that parameter estimates yielded by ML estimation may be biased to some extent, but, importantly, not by as much as when using mean imputation or case deletion [33, 34]. Finally, we were not able to provide standardized psychopathology scores and for symptom severity control we referred therefore to hospitalization demographics, which are less reliable than psychopathology scores. However, most patients were at least in partial remission for positive symptoms at the time of testing, which was the requirement for attending the routine neuropsychological testing and was controlled by therapists. We acknowledge that higher ADD and CDD could also mean that patients were suffering from more severe schizophrenic symptoms. Consequently, when interpreting the correlations between neuropsychological testing and equivalent doses, illness severity could be a major confounding factor. Furthermore, the medication regime while in hospital could be a confounding factor. While patients receiving sedatives as regularly prescribed medication were excluded from our study, inpatients may have received sedatives and antidepressants on an “on demand” basis that were not controlled for and could potentially worsen test scores. Additional research will be needed to determine further, for example, the clinical implications of our study and, in particular, the effects on different cognitive domains of clozapine's anticholinergic properties and its interaction with other neurotransmitter systems.

## Figures and Tables

**Table 1:** Demographics of the initial sample (*N* = 126), results represented by mean value and standard deviation or absolute frequencies.

Age (years)	28.2 (8.6)
Gender	
*N* female/*N* male	47/79
Educational level	
*N* none/*N* high school/*N* university/*N* unknown	56/47/18/5
Time since onset of illness (months)	21.2 (42.6)

**Table 2:** Dose equivalences for antipsychotic (RIS-Eq/CPZ-Eq) and anticholinergic (BZT-Eq) medication.

Drug	BZT-Eq 1 mg	RIS-Eq 1 mg	CPZ Eq 50 mg	Number of patients
Amitriptyline	10	*∗∗*	0	1
Amisulpride	0	*∗∗*	100	19
Aripiprazole	*∗∗*	*∗∗*	7,5	1
Benztropine	1	*∗∗*	*∗∗*	1
Biperiden	0,7	*∗∗*	*∗∗*	11
Clozapine	8	70	*∗∗*	27
Doxepin	41	*∗∗*	*∗∗*	1
Escitalopram	0	*∗∗*	*∗∗*	5
Lamotrigine	0	0	0	2
Lithium	0	0	0	5
Nortriptyline	73	*∗∗*	*∗∗*	1
Promethazine	367	*∗∗*	100	2
Olanzapine	17	5	*∗∗*	30
Quetiapine	733	140	*∗∗*	23
Risperidone	0	1/7,5^*∗*^	2	18
Valproate	0	0	0	6
Venlafaxine	0	*∗∗*	*∗∗*	1
Ziprasidone	0	*∗∗*	60	1

Note: ^*∗*^for risperidone injection, ^*∗∗*^no data. Source: [12, 31].

**(a):** 

Test parameter	Component loadings (VM)
VLMT (immediate recall)	.797
VLMT (interference list recall)	.805
VLMT (delayed recall)	.871
VLMT (sum lists 1 to 5)	.938
VLMT (delayed recall 2)	.893

VM: declarative verbal memory; VLMT: verbal learning and memory test.

**(b):** 

Variable	Component loadings			
	CVT	IPS	EF	ATT
RWT (formal lexical)	**.751**	−.023	−.095	−.011
RWT (semantic)	**.562**	−.166	−.172	−.253
WMS (text reproduction immediate)	**.922**	.048	.158	−.037
WMS (text reproduction delayed)	**.904**	−.015	.155	−.015
WMS (digit span forward)	.406	.020	**−.531**	.176
WMS (digit span backward)	**.450**	−.200	−.328	.269
TAP (divided attention IPS auditory)	.177	**.867**	−.320	.022
TAP (divided attention IPS visual)	−.163	**.699**	−.078	−.114
TAP (shifted reaction IPS)	−.109	**.676**	.106	.189
Trails A (time until completion)	−.232	**.465**	.313	−.040
Trails B (time until completion)	−.347	.362	**.400**	−.202
d2 (summation number)	.216	**−.585**	.034	.109
d2 (error percentage)	−.155	.027	.044	**.911**
WIE (number-symbol test)	−.167	**−.660**	−.480	−.067
TAP (shifted error reaction amount)	.138	−.148	**.890**	.100

Note: higher IPS and EF factor scores indicate lower performance because error amount and time parameters served as variables in the PCA (which becomes clear by examining the loading pattern of these components).

**(c):** 

Component	CVT	IPS	EF	ATT
CVT	1			
IPS	−.360	1		
EF	−.263	.179	1	
ATT	.091	−.042	−.018	1

CVT: complex verbal tasks; IPS: information processing speed; EF: executive functioning; ATT: attention; RWT: Regensburg Word Fluency Test; WMS: Wechsler Memory Scale; TAP: Test of Attentional Performance; TMT: Trail Making Test; d2: d2 Test of Attention; WIE: Wechsler intelligence test for adults; VLMT results were excluded and formed a separate domain (VM); see Table 3(a). Solution converged in 12 iterations.

**(d):** 

Variable	*N*	Mean PR	SD
VLMT sum scores	115	46,87	32,64
RWT (formal lexical)	109	31,71	27,06
RWT (semantic)	110	28,84	26,54
WMS (text reproduction immediate)	101	34,83	31,35
WMS (text reproduction delayed)	99	33,30	31,37
WMS (digit span forward)	100	45,69	29,43
WMS (digit span backward)	96	43,18	31,17
TAP (divided attention IPS auditory)	113	22,24	22,82
TAP (divided attention IPS visual)	23	35,48	25,37
TAP (shifted reaction IPS)	92	23,38	23,53
Trails A (time until completion)	116	44,81	28,74
Trails B (time until completion)	113	45,94	28,32
d2 (summation number)	101	27,64	28,53
d2 (error percentage)	101	42,98	29,64
WIE (number-symbol test)	61	33,04	24,98
TAP (shifted error reaction amount)	92	49,52	29,37

Note: the expected normal value for control population is a percentile rank (PR) of 50. Values below 50 are considered worse performance than expected for controls, pointing to cognitive impairment.

**Table 4:** Demographics of subgroup A, subgroup B, and the unmedicated patients group, results represented by mean value and standard deviation or absolute frequencies.

Variable	Subgroup A (*N* = 41)	Subgroup B (*N* = 63)	Unmedicated (*N* = 22)
Age (years)	26.7 (7.2)	29.6 (9.1)	26.7 (9.1)
Gender			
*N* female/*N* male	16/25	23/40	8/14
Educational level			
*N* none/*N* high school/*N* university/*N* unknown	22/12/6/1	25/24/10/4	9/11/2/0
Time since onset of illness (months)	12.9 (19.0)	31.7 (54.9)	3.8 (7.1)
